# Determining optimal strategies for primary prevention of cardiovascular disease: systematic review, cost-effectiveness review and network meta-analysis protocol

**DOI:** 10.1186/s13643-020-01366-x

**Published:** 2020-05-07

**Authors:** Olalekan A. Uthman, Lena Al-Khudairy, Chidozie U. Nduka, Rachel Court, Hema Mistry, G . J. Melendez-Torres, Sian Taylor-Phillips, Aileen Clarke

**Affiliations:** 1 Warwick-Centre for Applied Health Research and Delivery (WCAHRD), Division of Health Sciences, Warwick Medical School, University of Warwick, Coventry, CV4 7AL UK; 2 Peninsula Technology Assessment Group (PenTAG), College of Medicine and Health, University of Exeter, Exeter, England

## Abstract

**Background:**

Despite recent improvements in the burden of cardiovascular disease (CVD) in the UK, deaths from CVD are relatively high compared with other high-income countries. An estimated 7 million people in the UK are living with CVD, and the healthcare cost is approximately £11 billion annually. In more than 90% of cases, the risk of a first heart attack is thought to be related to modifiable risk factors including smoking, poor diet, lipidemia, high blood pressure, inactivity, obesity and excess alcohol consumption. The aim of the study is to synthesise evidence for the comparative effectiveness and cost-effectiveness of different interventions for the primary prevention of CVD.

**Methods:**

We will systematically search databases (for example, MEDLINE (Ovid), Embase (Ovid), Cochrane Library) and the reference lists of previous systematic reviews for randomised controlled trials that assess the effectiveness and cost-effectiveness of any form of intervention aimed at adult populations for the primary prevention of CVD, including but not limited to lipid lowering medications, blood pressure lowering medications, antiplatelet agents, nutritional supplements, dietary interventions, health promotion programmes, physical activity interventions or structural and policy interventions. Interventions may or may not be targeted at high-risk groups. Publications from any year will be considered for inclusion. The primary outcome will be all cause mortality. Secondary outcomes will be cardiovascular diseases related mortality, major cardiovascular events, coronary heart disease, incremental costs per quality-adjusted life years gained. If data permits, we will use network meta-analysis to compare and rank effectiveness of different interventions, and test effect modification of intervention effectiveness using subgroup analyses and meta-regression analyses.

**Discussion:**

The results will be important for policymakers when making decisions between multiple possible alternative strategies to prevent CVD. Compared to results from existing multiple separate pairwise meta-analyses, this overarching synthesis of all relevant work will enhance decision-making. The findings will be crucial to inform evidence-based priorities and guidelines for policies and planning prevention strategies of CVD.

**Systematic review registration:**

PROSPERO CRD42019123940.

## Background

Cardiovascular disease (CVD) includes all the diseases of the heart and circulation including coronary heart disease (CHD) and stroke. CVD accounts for the highest proportion of non-communicable disease deaths, resulting in 160,000 deaths in the UK annually [1–3]. Cardiovascular risk is determined by a variety of ‘upstream’ factors (such as healthy food production and availability, access to a safe environment that encourages physical activity and access to health education) as well as ‘downstream’ behavioural issues (such as unhealthy diet, smoking and physical inactivity). In more than 90% of cases, the risk of a first heart attack is related to nine potentially modifiable risk factors [4, 5]: smoking/tobacco use, poor diet, high blood cholesterol, high blood pressure, high blood glucose, insufficient physical activity, overweight/obesity, diabetes, psychosocial stress and excess alcohol consumption. A significant proportion of CVD morbidity and mortality can be prevented through population strategies for primary prevention. There is a major potential population health impact of improving our understanding of CVD prevention.

**Fig. 1:**
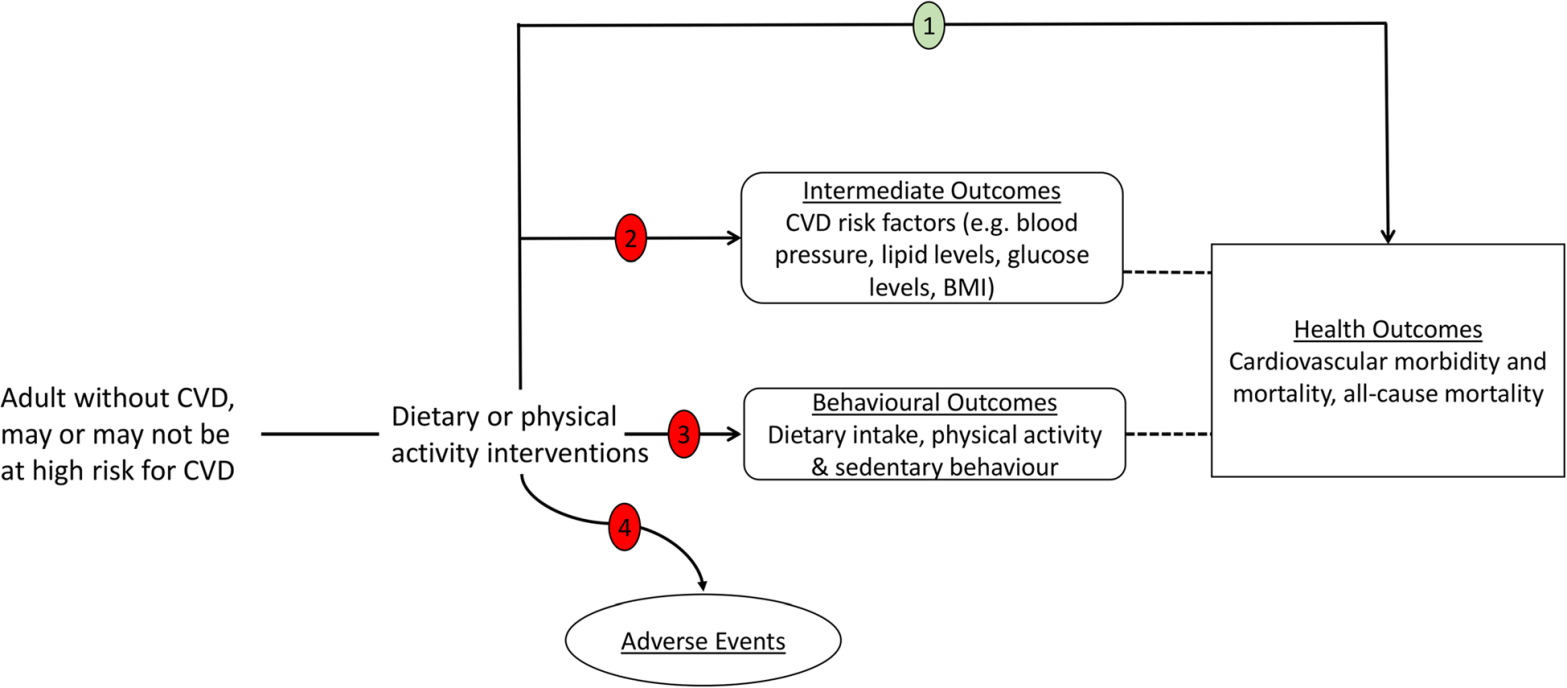
Analytic framework Note: pathway 1 will be systematically reviewed (in green), while pathways 2, 3 and 4 will not be reviewed (in red)

Though there are many pairwise systematic reviews and meta-analyses that have examined the effectiveness of drug, lifestyle and policy/structural interventions either separately and collectively (Additional file 1); there is no systematic review to date that has comprehensively synthesised all available evidence to understand the comparative effectiveness of these interventions for the primary prevention of CVD with the aim of supporting evidence-based recommendations to policymakers. The overarching aim of the proposed study is to fill this research gap by synthesising evidence for the comparative effectiveness of different interventions for the primary prevention of CVD using a network meta-analysis. The specific objectives are as follows: (1) to use comprehensive searches and to describe the scale and range of interventions that have been conducted and to categorise interventions and their components, (2) to determine which interventions, have the greatest probability of effectiveness for the primary prevention of CVD (see Fig. 1), (3) to identify which intervention components are associated with the greatest effectiveness for the primary prevention of CVD, (4) to examine reliability and conclusiveness of the available evidence on interventions for the primary prevention of CVD and to identify the areas with most potential benefit for future research, (5) to identify trial characteristics associated with prevention effect estimates, (6) to identify, appraise and synthesise any published economic evaluations and economic models of interventions for the primary prevention of CVD and (7) to determine the applicability and generalisability of interventions and the assessments of their cost-effectiveness to the UK NHS setting.

## Methods

The present protocol has been registered within the PROSPERO database (registration number CRD420119123940) and is reported in accordance with the Preferred Reporting Items for Systematic review and Meta-Analysis Protocols (PRISMA-P) checklist [6], and the PRISMA extension statement for reporting of systematic reviews incorporating network meta-analyses of healthcare interventions [7] (see checklist in Additional file 2). The record in PROSPERO and subsequent publications will be updated with any amendment made to the protocol.

### Eligibility criteria

We will evaluate each identified study against the following selection criteria:

Study population: adult populations (≥18 years of age) included in population-based studies, which may or may not be targeted at moderate/high CVD risk groups (such as hypertension, obesity, hyperlipidaemia, type 2 diabetes or a combination of these). As the review focuses on the primary prevention of CVD, we will exclude trials that included those who have experienced a previous myocardial infarction (MI), stroke, revascularisation procedure (coronary artery bypass grafting (CABG) or per cutaneous transluminal coronary angioplasty (PTCA)), and those with angina or angiographically defined coronary heart disease (CHD). Studies with mixed populations, i.e. both population with and without CVD will be included if data for the relevant primary prevention can be extracted.

Intervention: any form of intervention aimed at the primary prevention of CVD, including but not limited to drugs (lipid lowering medications, blood pressure lowering medications, antiplatelet agents), diet (nutritional supplements, dietary interventions), physical activity or public health (health promotion programmes, structural and policy interventions) (Table 1).

**Table 1:** Health technologies (interventions)

Pharmacologic interventions			
Lipid lowering medications	Blood pressure lowering medications	Nutritional supplements	Others
Atorvastatin Fluvastatin Lovastatin Pitavastatin Pravastatin Rosuvastatin Fenofibrate Bezafibrate Ezetimibe	ACE inhibitors Angiotensin receptor blockers (ARBs) Calcium channel blockers Thiazide diuretics Adrenergic receptor antagonists (alpha and beta blockers) Vasodilators Renin inhibitors	Vitamin D, E, K and multivitamins Niacin Omega 3 and fatty acids Anti-oxidants Calcium Co-enzyme Q10 Selenium Folic acid Garlic	Fixed dose combinations ‘polypill’ Antiplatelet agent (Aspirin)
Lifestyle-modification interventions			
Dietary interventions	Health promotion	Exercise/physical activity in general	
Mediterranean diet Fibres Nut consumption Chocolate Fruits and vegetables Green and black tea Reduced salt intake Reduced fat intake	Smoking cessation Weight reduction Reduction in alcohol intake Multiple risk factors intervention Digital health promotion	Endurance (or aerobic) exercise Strengthening exercise Balance Tai-chi Flexibility Yoga Aquatic Qiqong Transcendental meditation Combined exercise	
Structural and policy-based interventions (population-wide interventions)			
Taxation and subsidies Mass media campaigns Food and menu labelling Local food environment Worksite wellness programmes Marketing restrictions Quality standards Healthy local environment Addressing air pollution			

Comparators: other forms of intervention (such as a minimal intervention, active intervention, concomitant intervention), placebo, usual care or no intervention control group or wait list control.

Outcome measures: The primary outcome will be all cause mortality. Secondary outcomes will be cardiovascular diseases related mortality, major cardiovascular events (defined as fatal and non-fatal myocardial infarction, sudden cardiac death, revascularisation, fatal and non-fatal stroke and fatal and non-fatal heart failure), coronary heart disease (fatal and non-fatal myocardial infarction and sudden cardiac death, excluding silent myocardial infarction), incremental costs per quality-adjusted life years gained reported alongside a randomised trial.

Study design: randomised controlled trials (RCTs) of at least 6 months’ duration of follow-up. Units of randomisation could be either individuals or clusters (such as family, workplace).

### Information sources and search strategy

#### Clinical effectiveness

Due to the likelihood of a high volume of relevant trials to be included, we will follow standard guidelines for integrating existing systematic reviews into new reviews [8, 9]. Where existing systematic reviews with acceptable search and study selection methods (especially Cochrane reviews) are available for any of the intervention categories, these will be used as a starting point to identify relevant studies. Initial searches for relevant systematic reviews will not be restricted by date. Searches will not be restricted by language.

a) A comprehensive literature search for existing systematic reviews will be developed iteratively and undertaken in major medical and health-related electronic bibliographic databases including MEDLINE (Ovid), Embase (Ovid), Cochrane Database of Systematic Reviews (Wiley) and DARE (CRD). Systematic reviews that potentially include primary studies meeting our inclusion criteria will be selected. Records for the included (and, if available, excluded) studies in all selected systematic reviews will be identified and imported into EndNote using HubMed Citation Finder [10], systematically de-duplicated and screened.

The most recent systematic review for any intervention or intervention category will be assessed using AMSTAR-2 [11] items 4, 5 and 7 to help determine whether or not the search and study identification methods are acceptable for the purpose of identifying studies for this network meta-analysis. If not, the next most recent review where available will be assessed using the same criteria. An analysis of the search dates of the chosen reviews will inform the date limit used for the search for more recent trials (i.e. those that are not yet included in a published review). The aim of this is to ensure all time periods are covered.

b) The search for recent trials will be developed iteratively and will be informed by records of a broad cross-section of known studies. Searching based on the concepts of prevention and CVD outcomes, or on intervention terms and CVD outcomes, will be considered and tested. Scoping searches have retrieved very high numbers of trials in Cochrane Controlled Register of Trials (CENTRAL) (Wiley) using either approach. Our trial search will focus on CENTRAL (Wiley). For any interventions where no acceptable systematic review is available, a search for trials with no date limit and including relevant intervention terms will be performed. A draft search strategy in CENTRAL (Wiley) is available in Additional file 3**.**

c) In order to capture studies with more obscure records and those that may have been excluded by previous systematic reviews, we will run a highly sensitive search with no date limit and use machine learning to identify a proportion of these records for screening (see ‘Selection Process’ below).

d) Finally, the reference lists of included studies will be examined for additional relevant studies.

#### Cost-effectiveness

We will develop searches iteratively, referring to known articles, existing strategies and assessed search filters [12]. Search terms will include economic, cost and health-related quality of life-related terms combined with CVD terms. Other concepts may be added as necessary. Databases will include the following: MEDLINE (Ovid), MEDLINE In-Process Citations and Daily Update (Ovid) and Embase (Ovid). Searches will be limited to studies in the English language, and to humans. We will also check the reference lists of included studies and any relevant reviews.

### Selection process

In order to reduce the workload of screening the searches result from the highly sensitive search with no date limit, we will develop a bespoke classifier/algorithm to identify potentially relevant studies. We will aim to achieve a high-performing algorithm comparable to human screening [13]. The computer will be fed with training data using the included and excluded studies found via our other searches. From this algorithm, the machine can make predictions (include or exclude) on other titles and abstracts that it has never seen. We will screen the titles/abstracts of a small proportion 10% of these results.

### Data collection process

Data will be independently extracted using a pre-specified piloted proforma by two reviewers, with discrepancies resolved by a third reviewer. We will use a data collection form for study characteristics and outcome data. One author will extract study characteristics from the included studies and a second author will spot-checked study characteristics for accuracy against the trial report. Any inconsistencies will be resolved by discussion.

We will extract the following characteristics:

Study citation: year(s) of study, registration number to trial registries, year of publication, location, setting, number of centres, sample size, diagnostic criteria, funding/sponsor

Methods: including study design (type of RCT), number of arms, risk of bias (see below)

Participants: number, mean age, age range, gender, severity of condition, diagnostic criteria, baseline measures of physiological functioning (e.g. cardiovascular function, blood pressure, body mass index, blood glucose, HbA1C, smoking history), inclusion and exclusion criteria

Interventions: intervention, comparison, concomitant medications and excluded medications

Outcomes: primary and secondary outcomes specified and collected, and time points reported including information on whether an intention to treat approach has been used and how it was defined

### Data items and measurement of treatment effect

#### Clinical effectiveness

We will report dichotomous outcomes as risk ratios (RRs). For continuous outcomes, we will calculate mean differences (MDs) when the studies report homogenous outcomes. Time-to-event outcomes or generic inverse variance outcomes will be expressed as the logarithm of hazard ratio (HR).

If possible, we will use the intention-to-treat population for all analyses. When effect sizes are incompletely reported, we will contact the corresponding author. When the SDs of absolute changes from baseline are not available from individual trials, we will impute them as described in detail in the Cochrane Handbook [14]. In brief, we will assume a correlation of *r* = 0.5 between baseline and follow-up to estimate SD for change from baseline. Using the imputed correlation coefficient values, we will calculate SDs for the change from baseline for the studies with missing SDs using the following formula:

$$ {\mathrm{SD}}_{\mathrm{change}}=\sqrt{{{\mathrm{SD}}^2}_{\mathrm{baseline}}+{{\mathrm{SD}}^2}_{\mathrm{final}}-\left(2\ast r\ast {\mathrm{SD}}_{\mathrm{baseline}}\ast {\mathrm{SD}}_{\mathrm{final}}\right)} $$

We will include cluster-randomised trials in the meta-analysis along with individually randomised trials (unit of analysis issues). Cluster-randomised trials will be labelled with a (C). For cluster-randomised trials to be included in the network meta-analyses, we will adjust for design effect using an ‘approximation method’ [15] if the trial did not use a cluster-adjusting analytical strategy. The ‘approximation method’ entails calculation of an ‘effective sample size’ for the comparison groups by dividing the original sample size by the ‘design effect’, which is 1 + (M − 1) ICC, where M is the average cluster size and ICC is the intra-cluster correlation coefficient. For dichotomous data, we will divide both the number of participants and the number who experience the event by the same design effect, while for continuous data, only the sample size will be reduced (means and standard deviations (SDs) will be left unchanged).

#### Cost-effectiveness

For each identified study that meets the selection criteria, we will extract the following data: country, study design, population, intervention(s), comparator(s), type of economic analysis, perspective, model type (structure and key assumptions), time horizon, effectiveness data, primary outcome, resource use and unit cost data, price year, discounting and the results of the base-case and sensitivity analyses. Data such as outcomes and characteristics will be synthesised quantitatively, where appropriate or narratively. For the primary outcome, the preferred measure will be cost per quality-adjusted life years (QALY) gained.

### Geometry of the network

A network plot will be created to describe and present the geometry of the intervention network of comparisons across trials [16]. Intervention comparisons not connected to the rest of the network will be excluded from the network meta-analysis and describe that comparison separately. In the network diagram, each node represents an intervention, and the edges represent head-to-head comparisons between a pair of interventions. The size of a node reflects the sample size for the intervention, and the thickness of an edge reflects the number of trials that included the comparison [16].

### Risk of bias in individual studies

We will use the Cochrane Collaboration’s tool for assessing risk of bias for quality assessment of the included trials at trial level [17]. The trials were graded (unclear, high or low risk of bias) based on sequence generation, allocation concealment, blinding of outcome assessor, incomplete outcome data and selective outcome reporting. Risk of bias for the included trials will be assessed using the Robot Reviewer [18]. We will use the quality assessment of economic modelling checklist developed by Philips et al. [19] to assess the quality of the economic evaluation studies. We will use the Consolidated Health Economic Evaluation Reporting Standards (CHEERS) checklist [20] to assess the quality of the economic evaluation studies

For cluster-randomised trials, we will assess the following cluster-specific risks of bias as outlined in the Cochrane Handbook for Systematic Reviews of Interventions [14].

Recruitment bias - whether the individuals participating in the trial were blinded to the type of cluster they were in before agreeing to participate

Baseline imbalance - whether there were differences in baseline characteristics between the randomised groups

Loss of clusters - whether any complete clusters were lost to follow-up and the reasons

Incorrect analysis - whether the proper statistical analysis was carried out for a cluster-randomised design

Comparability with individually randomised trials - whether the cluster-randomisation method could have resulted in different intervention effects than an individually randomised trial

When considering treatment effects, we will take into account the risk of bias of the studies that contribute to that outcome.

### Planned methods of analysis

#### Characteristics of the included studies

We will produce descriptive statistics and study population characteristics across all eligible trials, describing the types of comparisons and other clinical or methodological variables, such as age, co-medication, country and study follow-up period.

#### Pairwise meta-analyses

In the first step we will perform series of conventional pairwise random-effects meta-analyses by combining studies that compared the same interventions, including the comparison between active treatments and the different control arms. If very few trials are available or the requirements of network meta-analysis are not met, it can be that network meta-analysis will not be appropriate and, in this case, conventional pairwise meta-analysis will be applied.

#### Assessment of heterogeneity

We will assess between-trials heterogeneity (variability in relative treatment effects within the same treatment comparison) using the tau-squared (the variance of the random effects distribution). The heterogeneity variance will be assumed common across the various treatment comparisons (grouped by comparison type) and we will compare the empirical distribution with predictive distributions [21–23]. We will explore the potential reasons for heterogeneity by subgroup analysis described below.

#### Assessment of the transitivity assumption

The transitivity assumption will be assessed by investigating the distribution of clinical and methodological variables that can act as effect modifiers across treatment comparisons [24].

We will assume that patients who fulfil the inclusion criteria are equally likely to be randomised to any of the interventions of interest (i.e. jointly randomisable). When additional evidence of intransitivity is lacking and potential effect modifiers have similar distributions across the included studies, network meta-analysis is likely to give valid results.

#### Network meta-analysis

We will conduct network meta-analyses [25, 26] to compare effectiveness of the different types of interventions for primary prevention of CVD. Given the substantial number of interventions and the limited evidence base available to construct the network of evidence, (in terms of both the number of trials and the number of direct comparisons between active interventions), we will use a two-level hierarchical network meta-analysis to borrow strength within the classes of intervention, strengthening inferences and potentially reducing the uncertainty around individual intervention effects. This will consequently increase our ability to rank these and to inform decision-making frameworks [27]. The two-level hierarchical network meta-analysis (level 1: intervention type and level 2: intervention class) will incorporate exchangeability between interventions of the same class to predict an effect estimate for each of the interventions individually [27].

We will calculate the probability of a given intervention having the largest beneficial effects as the proportion of simulations in which that intervention will be ranked as the ‘best’ according to the relative prevention effect estimate. In addition, we will calculate alternative rankings (second and third best, etc.) because in some policy and practice areas, the best intervention might be unavailable, too costly or contraindicated. Probability values will be summarised and reported as surface under the cumulative ranking (SUCRA) and graphically ranked using rankograms. SUCRA = 1 if an intervention always ranks first and SUCRA = 0 if it always ranks last.

All the network meta-analyses analyses [25, 26] will be conducted using a Markov chain Monte Carlo method. The models will account for the correlation between prevention effect estimates induced by three or more arm trials using the multi-arm trial code. At least two Markov chains will be run simultaneously using different arbitrary initial values. Convergence to a stable solution will be checked by viewing plots of the sampled simulations and using the Brooks-Gelman-Rubin diagnostic tool. All results will be reported as posterior medians of prevention effect estimate with corresponding 95% credible intervals (CrIs). Credible intervals are the Bayesian equivalent of classical confidence intervals. A 95% CrI can be interpreted as a 95% probability that the parameter takes a value in the specified range.

#### Assessment of inconsistency

The evaluation of transitivity will be supplemented with a statistical evaluation of consistency, the agreement between direct and indirect evidence. We will evaluate consistencies between direct and indirect comparisons in the network of evidence using the method of ‘node-splitting’ [28], by calculating the difference for each pair of interventions and the probability of whether direct estimates surpass the indirect estimate.

#### Investigation of heterogeneity and inconsistency

We anticipate several sources of heterogeneity relating to the content of the intervention and study design. We will test effect modification of intervention effectiveness using subgroup analyses and meta-regression analyses. For example, where there are sufficient data, we will stratify our analyses (subgroup) by the following: population risk groups (healthy versus high-risk), trial period (older versus recent), sex (male versus female) and age (young adult versus elderly population), by intervention components and by characteristics of outcome measures. Meta-regression analyses will be used to explore components of interventions, participant characteristics and outcome measure characteristics that can predict prevention effect estimates within and across different types of interventions. The network meta-regression will be performed by allowing for a common treatment-covariate interaction for each intervention in the network meta-analysis [29].

#### Publication bias

To assess small study effects and publication bias, we will use funnel plots of pairwise meta-analyses if 10 or more studies are included. We will also use a comparison-adjusted funnel plot for relative treatment effects between all active and control interventions [30].

#### Systematic review of cost-effectiveness studies

In order to make different incremental cost-effectiveness ratios (ICERs) comparable, we will convert them from their currencies to pounds sterling (£) using purchasing power parities [31]. Once converted to pounds sterling, the cost data will be inflated to 2019/20 prices using the Hospital and Community Health Services index [32]. For studies that do not report price year, the incremental cost-effectiveness ratios will be converted to pounds sterling using the published year of study as the assumed price year.

#### Statistical software

The analysis and presentation of results will be performed using R (meta, netmeta and BUGSnet packages) [33–36].

### Assessment of the confidence in the evidence from NMA

The confidence in the relative treatment effect estimated in network meta-analysis for the primary outcome will be evaluated using the Confidence in Network Meta-Analysis framework [37], implemented in the web application. This tool evaluates the credibility of the findings across the domains of within-study bias, across-study bias, indirectness, imprecision, heterogeneity and incoherence.

## Discussion

Despite recent improvements in the burden of cardiovascular disease in the UK, deaths from cardiovascular disease are relatively high compared with other high-income countries. There is a major potential population health impact of improving our understanding of CVD prevention. Identifying the most effective intervention however remains a challenge for researchers and policymakers.

This study will provide highly relevant findings using innovative methods to determine optimal strategies for primary prevention of CVD. The results will be important for policymakers when making decisions between the multiple possible alternative strategies to prevent CVD. Compared to results from the existing multiple separate pairwise meta-analyses, this overarching summary of all relevant work will enhance decision-making. The findings will be crucial to inform evidence-based priorities and guidelines for policies and planning prevention strategies. The public will have answers to which intervention or combination of interventions has the greatest probability of being most effective and most cost-effective in preventing CVD. Findings from our study will inform research funders and improve the targeting of subsequent research, and evaluation of additional population interventions.

Given the paucity of randomised evidence on population-wide interventions, this project will assess data from observational studies which are more prone to potential bias. We will mitigate this by using Bayesian approach to coherently synthesise evidence from both randomised and non-randomised evidence, which will take into account the potential bias in the observational studies using an extra variance component.

Findings from this study will be internationally relevant. Our findings will be widely shared with academics (as journal articles); policymakers (such as The National Institute for Health and Care Excellence), stakeholders and key organisations such as the British Heart Foundation (executive summaries) and patient groups (lay summaries).

## Supplementary information

**Additional file 1.** List of existing of existing systematic reviews

**Additional file 2.** PRISMA-P Checklist

**Additional file 3.** Cochrane CENTRAL search strategy

## Data Availability

Not applicable since all data that are referred to in this article will have been obtained through reading original studies or contacting the authors of cited studies.

## Abbreviations

AMSTAR: A MeaSurement Tool to Assess systematic Reviews
CABG: Coronary artery bypass grafting
CEA: Cost-effectiveness analysis
CHD: Coronary heart disease
CHEERS: Consolidated Health Economic Evaluation Reporting Standards
CRD: Centre for Reviews and Dissemination
CVD: Cardiovascular disease
HR: Hazard ratio
HTA: Health technology assessment
ICC: Intra-cluster correlation
ICER: Incremental cost-effectiveness ratio
MD: Mean difference
MI: Myocardial infarction
NHS: National Health Scheme
NIHR: National Institute for Health Research
NMA: Network meta-analysis
PTCA: Percutaneous transluminal coronary angioplasty
QALY: Quality-adjusted life year
RCT: Randomised controlled trial
RR: Risk ratio
SD: Standard deviation
SUCRA: Surface under the cumulative ranking
TSA: Trial Sequential Analysis
