# Supplementary material for: Determining optimal strategies for primary prevention of cardiovascular disease: systematic review, cost-effectiveness review and network meta-analysis protocol
Source: Syst Rev. 2020 May 7;9:105. doi: 10.1186/s13643-020-01366-x (PMC7204030; doi:10.1186/s13643-020-01366-x)
Supplement: Supplementary file 1 — Additional file 1. List of existing of existing systematic reviews [file 13643_2020_1366_MOESM1_ESM.docx]

**Appendix 1: Existing Systematic Reviews[1-249]**

1. Wikstrand J: **Primary prevention with beta-blockade in patients with hypertension: review of results and clinical implications**. *J Cardiovasc Pharmacol* 1990, **16 Suppl 5**:S64-75.

2. Brunner E, White I, Thorogood M, Bristow A, Curle D, Marmot M: **Can dietary interventions change diet and cardiovascular risk factors? A meta-analysis of randomized controlled trials**. *Am J Public Health* 1997, **87**(9):1415-1422.

3. Crouse JR, 3rd, Byington RP, Furberg CD: **HMG-CoA reductase inhibitor therapy and stroke risk reduction: an analysis of clinical trials data**. *Atherosclerosis* 1998, **138**(1):11-24.

4. Warshafsky S, Packard D, Marks SJ, Sachdeva N, Terashita DM, Kaufman G, Sang K, Deluca AJ, Peterson SJ, Frishman WH: **Efficacy of 3-hydroxy-3-methylglutaryl coenzyme A reductase inhibitors for prevention of stroke**. *Journal of General Internal Medicine* 1999, **14**(12):763-774.

5. Clarke R, Armitage J: **Vitamin supplements and cardiovascular risk: review of the randomized trials of homocysteine-lowering vitamin supplements**. *Semin Thromb Hemost* 2000, **26**(3):341-348.

6. Hart RG, Halperin JL, McBride R, Benavente O, Man-Son-Hing M, Kronmal RA: **Aspirin for the primary prevention of stroke and other major vascular events: meta-analysis and hypotheses**. *Arch Neurol* 2000, **57**(3):326-332.

7. Ketola E, Sipila R, Makela M: **Effectiveness of individual lifestyle interventions in reducing cardiovascular disease and risk factors**. *Ann Med* 2000, **32**(4):239-251.

8. Pignone M, Phillips C, Mulrow C: **Use of lipid lowering drugs for primary prevention of coronary heart disease: meta-analysis of randomised trials**. *Bmj* 2000, **321**(7267):983-986.

9. Quan A, Kerlikowske K, Gueyffier F, Boissel JP, investigators I: **Pharmacotherapy for hypertension in women of different races**. *Cochrane Database Syst Rev* 2000(3):CD002146.

10. Hooper L, Summerbell CD, Higgins JP, Thompson RL, Capps NE, Smith GD, Riemersma RA, Ebrahim S: **Dietary fat intake and prevention of cardiovascular disease: systematic review**. *Bmj* 2001, **322**(7289):757-763.

11. Huang ES, Meigs JB, Singer DE: **The effect of interventions to prevent cardiovascular disease in patients with type 2 diabetes mellitus**. *Am J Med* 2001, **111**(8):633-642.

12. Li W, Gueyffierd F, Liu GZ, Zhang YQ, Liu LS: **The effect of antihypertensive treatment on cardiovascular events--a meta-analysis of four clinical trials in China**. *Biomed Environ Sci* 2001, **14**(4):341-349.

13. Sanmuganathan PS, Ghahramani P, Jackson PR, Wallis EJ, Ramsay LE: **Aspirin for primary prevention of coronary heart disease: safety and absolute benefit related to coronary risk derived from meta-analysis of randomised trials**. *Heart* 2001, **85**(3):265-271.

14. Antithrombotic Trialists C: **Collaborative meta-analysis of randomised trials of antiplatelet therapy for prevention of death, myocardial infarction, and stroke in high risk patients.[Erratum appears in BMJ 2002 Jan 19;324(7330):141]**. *Bmj* 2002, **324**(7329):71-86.

15. Hayden M, Pignone M, Phillips C, Mulrow C: **Aspirin for the primary prevention of cardiovascular events: a summary of the evidence for the U.S. Preventive Services Task Force.[Summary for patients in Ann Intern Med. 2002 Jan 15;136(2):I55; PMID: 11928737]**. *Annals of internal medicine* 2002, **136**(2):161-172.

16. Bredie SJ, Wollersheim H, Verheugt FW, Thien T: **Low-dose aspirin for primary prevention of cardiovascular disease**. *Semin Vasc Med* 2003, **3**(2):177-184.

17. Eidelman RS, Hebert PR, Weisman SM, Hennekens CH: **An update on aspirin in the primary prevention of cardiovascular disease**. *Archives of Internal Medicine* 2003, **163**(17):2006-2010.

18. Law M, Wald N, Morris J: **Lowering blood pressure to prevent myocardial infarction and stroke: a new preventive strategy**. *Health Technology Assessment* 2003, **7**(31).

19. Morris CD, Carson S: **Routine Vitamin Supplementation To Prevent Cardiovascular Disease: A Summary of the Evidence for the U.S. Preventive Services Task Force**. *Annals of internal medicine* 2003, **139**(1):56.

20. Staessen JA, Wang JG, Thijs L: **Cardiovascular prevention and blood pressure reduction: a quantitative overview updated until 1 March 2003**. *J Hypertens* 2003, **21**(6):1055-1076.

21. Carlberg B, Samuelsson O, Lindholm LH: **Atenolol in hypertension: is it a wise choice?** *The Lancet* 2004, **364**(9446):1684-1689.

22. Eidelman RS, Hollar D, Hebert PR, Lamas GA, Hennekens CH: **Randomized Trials of Vitamin E in the Treatment and Prevention of Cardiovascular Disease**. *Archives of Internal Medicine* 2004, **164**(14):1552.

23. Harris GD, White RD: **Lifestyle modifications for the prevention and treatment of cardiovascular disease: an evidence-based approach**. *Mo Med* 2004, **101**(3):222-226.

24. Hooper L, Bartlett C, Davey SG, Ebrahim S: **Advice to reduce dietary salt for prevention of cardiovascular disease**. *Cochrane Database Syst Rev* 2004(1):Cd003656.

25. Hooper L, Thompson RL, Harrison RA, Summerbell CD, Moore H, Worthington HV, Durrington PN, Ness AR, Capps NE, Davey Smith G *et al*: **Omega 3 fatty acids for prevention and treatment of cardiovascular disease**. *Cochrane Database Syst Rev* 2004(4):Cd003177.

26. Kubler W: **[Primary prevention of coronary heart disease with aspirin]**. *Z Kardiol* 2004, **93 Suppl 2**:II33-36.

27. Shekelle PG, Morton SC, Jungvig LK, Udani J, Spar M, Tu W, Suttorp MJ, Coulter I, Newberry SJ, Hardy M: **Effect of supplemental vitamin E for the prevention and treatment of cardiovascular disease**. *Journal of General Internal Medicine* 2004, **19**(4):380-389.

28. Miller ER, Pastor-Barriuso R, Dalal D, Riemersma RA, Appel LJ, Guallar E: **Meta-Analysis: High-Dosage Vitamin E Supplementation May Increase All-Cause Mortality**. *Annals of internal medicine* 2005, **142**(1):37.

29. Pham DQ, Plakogiannis R: **Vitamin E supplementation in cardiovascular disease and cancer prevention: Part 1**. *Ann Pharmacother* 2005, **39**(11):1870-1878.

30. Allemann S, Diem P, Egger M, Christ ER, Stettler C: **Fibrates in the prevention of cardiovascular disease in patients with type 2 diabetes mellitus: meta-analysis of randomised controlled trials**. *Curr Med Res Opin* 2006, **22**(3):617-623.

31. Berger JS, Roncaglioni MC, Avanzini F, Pangrazzi I, Tognoni G, Brown DL: **Aspirin for the primary prevention of cardiovascular events in women and men: a sex-specific meta-analysis of randomized controlled trials**. *Jama* 2006, **295**(3):306-313.

32. Costa J, Borges M, David C, Carneiro AV: **Efficacy of lipid lowering drug treatment for diabetic and non-diabetic patients: metaanalysis of randomised controlled trials**. *Bmj* 2006, **332**(7550):1115-1124.

33. Curioni C, Andre C, Veras R: **Weight reduction for primary prevention of stroke in adults with overweight or obesity**. *Cochrane Database of Systematic Reviews* 2006(4):CD006062.

34. Ding EL, Hutfless SM, Ding X, Girotra S: **Chocolate and prevention of cardiovascular disease: a systematic review**. *Nutr Metab (Lond)* 2006, **3**:2.

35. Rosian I, Pichlbauer E, Stuerzlinger H: **The use of statins in primary prevention (Structured abstract)**. In: *Health Technology Assessment Database.* German Agency for Health Technology Assessment at the German Institute for Medical Documentation and Information (DAHTA DIMDI); 2006.

36. Salpeter SR, Walsh JME, Greyber E, Salpeter EE: **Brief report: Coronary heart disease events associated with hormone therapy in younger and older women**. *Journal of General Internal Medicine* 2006, **21**(4):363-366.

37. Thavendiranathan P, Bagai A, Brookhart MA, Choudhry NK: **Primary prevention of cardiovascular diseases with statin therapy: a meta-analysis of randomized controlled trials**. *Archives of Internal Medicine* 2006, **166**(21):2307-2313.

38. Ali R, Alexander KP: **Statins for the primary prevention of cardiovascular events in older adults: a review of the evidence**. *Am J Geriatr Pharmacother* 2007, **5**(1):52-63.

39. Cahill K, Stead LF, Lancaster T: **Nicotine receptor partial agonists for smoking cessation**. In: *Cochrane Database of Systematic Reviews.* edn.: John Wiley & Sons, Ltd; 2007.

40. Campbell CL, Smyth S, Montalescot G, Steinhubl SR: **Aspirin dose for the prevention of cardiovascular disease: a systematic review**. *Jama* 2007, **297**(18):2018-2024.

41. Clarke R, Lewington S, Sherliker P, Armitage J: **Effects of B-vitamins on plasma homocysteine concentrations and on risk of cardiovascular disease and dementia**. *Curr Opin Clin Nutr Metab Care* 2007, **10**(1):32-39.

42. Dahlof B, Devereux RB, Kjeldsen SE, Lyle PA, Zhang Z, Edelman JM: **Atenolol as a comparator in outcome trials in hypertension: a correct choice in the past, but not for the future?** *Blood Press* 2007, **16**(1):6-12.

43. Saha SA, Kizhakepunnur LG, Bahekar A, Arora RR: **The role of fibrates in the prevention of cardiovascular disease--a pooled meta-analysis of long-term randomized placebo-controlled clinical trials**. *American Heart Journal* 2007, **154**(5):943-953.

44. Estes K, Thomure J: **Aspirin for the primary prevention of adverse cardiovascular events**. *Crit Care Nurs Q* 2008, **31**(4):324-339.

45. Mills EJ, Rachlis B, Wu P, Devereaux PJ, Arora P, Perri D: **Primary prevention of cardiovascular mortality and events with statin treatments: a network meta-analysis involving more than 65,000 patients**. *Journal of the American College of Cardiology* 2008, **52**(22):1769-1781.

46. Sare GM, Gray LJ, Bath PMW: **Association between hormone replacement therapy and subsequent arterial and venous vascular events: a meta-analysis**. *European Heart Journal* 2008, **29**(16):2031-2041.

47. **Aspirin in the primary and secondary prevention of vascular disease: collaborative meta-analysis of individual participant data from randomised trials**. *The Lancet* 2009, **373**(9678):1849-1860.

48. Berger JS, Krantz MJ, Kittelson JM, Hiatt WR: **Aspirin for the prevention of cardiovascular events in patients with peripheral artery disease: a meta-analysis of randomized trials**. *Jama* 2009, **301**(18):1909-1919.

49. Brugts JJ, Yetgin T, Hoeks SE, Gotto AM, Shepherd J, Westendorp RGJ, de Craen AJM, Knopp RH, Nakamura H, Ridker P *et al*: **The benefits of statins in people without established cardiovascular disease but with cardiovascular risk factors: meta-analysis of randomised controlled trials**. *Bmj* 2009, **338**(jun30 1):b2376-b2376.

50. Calvin AD, Aggarwal NR, Murad MH, Shi Q, Elamin MB, Geske JB, Fernandez-Balsells MM, Albuquerque FN, Lampropulos JF, Erwin PJ *et al*: **Aspirin for the primary prevention of cardiovascular events: a systematic review and meta-analysis comparing patients with and without diabetes**. *Diabetes Care* 2009, **32**(12):2300-2306.

51. De Berardis G, Sacco M, Strippoli GF, Pellegrini F, Graziano G, Tognoni G, Nicolucci A: **Aspirin for primary prevention of cardiovascular events in people with diabetes: meta-analysis of randomised controlled trials**. *Bmj* 2009, **339**:b4531.

52. Law MR, Morris JK, Wald NJ: **Use of blood pressure lowering drugs in the prevention of cardiovascular disease: meta-analysis of 147 randomised trials in the context of expectations from prospective epidemiological studies**. *Bmj* 2009, **338**:b1665.

53. Wolff T: **Aspirin for the Primary Prevention of Cardiovascular Events: An Update of the Evidence for the U.S. Preventive Services Task Force**. *Annals of internal medicine* 2009, **150**(6):405.

54. **Efficacy and safety of more intensive lowering of LDL cholesterol: a meta-analysis of data from 170 000 participants in 26 randomised trials**. *The Lancet* 2010, **376**(9753):1670-1681.

55. Angermayr L, Melchart D, Linde K: **Multifactorial lifestyle interventions in the primary and secondary prevention of cardiovascular disease and type 2 diabetes mellitus--a systematic review of randomized controlled trials**. *Ann Behav Med* 2010, **40**(1):49-64.

56. Arguedas JA, Leiva V, Wright JM: **Blood pressure targets for hypertension in patients with diabetes mellitus**. In: *Cochrane Database of Systematic Reviews.* edn.: John Wiley & Sons, Ltd; 2010.

57. Bukkapatnam RN, Gabler NB, Lewis WR: **Statins for primary prevention of cardiovascular mortality in women: a systematic review and meta-analysis**. *Prev Cardiol* 2010, **13**(2):84-90.

58. Jun M, Foote C, Lv J, Neal B, Patel A, Nicholls SJ, Grobbee DE, Cass A, Chalmers J, Perkovic V: **Effects of fibrates on cardiovascular outcomes: a systematic review and meta-analysis**. *Lancet (London, England)* 2010, **375**(9729):1875-1884.

59. Leaberry BA: **Aspirin for the prevention of cardiovascular disease: systematic review**. *J Nurs Care Qual* 2010, **25**(1):17-21.

60. Loomba RS, Arora R: **Prevention of cardiovascular disease utilizing fibrates--a pooled meta-analysis**. *Am J Ther* 2010, **17**(6):e182-188.

61. Mora S, Glynn RJ, Hsia J, MacFadyen JG, Genest J, Ridker PM: **Statins for the primary prevention of cardiovascular events in women with elevated high-sensitivity C-reactive protein or dyslipidemia: results from the Justification for the Use of Statins in Prevention: An Intervention Trial Evaluating Rosuvastatin (JUPITER) and meta-analysis of women from primary prevention trials**. *Circulation* 2010, **121**(9):1069-1077.

62. Petretta M, Costanzo P, Perrone-Filardi P, Chiariello M: **Impact of gender in primary prevention of coronary heart disease with statin therapy: A meta-analysis**. *International Journal of Cardiology* 2010, **138**(1):25-31.

63. Ray KK: **Statins and All-Cause Mortality in High-Risk Primary Prevention**. *Archives of Internal Medicine* 2010, **170**(12):1024.

64. Saha SA, Arora RR: **Fibrates in the prevention of cardiovascular disease in patients with type 2 diabetes mellitus--a pooled meta-analysis of randomized placebo-controlled clinical trials**. *International Journal of Cardiology* 2010, **141**(2):157-166.

65. Wang L, Manson JE, Song Y, Sesso HD: **Systematic review: Vitamin D and calcium supplementation in prevention of cardiovascular events**. *Annals of internal medicine* 2010, **152**(5):315-323.

66. Younis N, Williams S, Ammori B, Soran H: **Role of aspirin in the primary prevention of cardiovascular disease in diabetes mellitus: a meta-analysis**. *Expert Opin Pharmacother* 2010, **11**(9):1459-1466.

67. Zhang C, Sun A, Zhang P, Wu C, Zhang S, Fu M, Wang K, Zou Y, Ge J: **Aspirin for primary prevention of cardiovascular events in patients with diabetes: A meta-analysis**. *Diabetes Res Clin Pract* 2010, **87**(2):211-218.

68. Astrup A, Dyerberg J, Elwood P, Hermansen K, Hu FB, Jakobsen MU, Kok FJ, Krauss RM, Lecerf JM, LeGrand P *et al*: **The role of reducing intakes of saturated fat in the prevention of cardiovascular disease: where does the evidence stand in 2010?** *Am J Clin Nutr* 2011, **93**(4):684-688.

69. Bangalore S, Kumar S, Lobach I, Messerli FH: **Blood Pressure Targets in Subjects With Type 2 Diabetes Mellitus/Impaired Fasting Glucose**. *Circulation* 2011, **123**(24):2799-2810.

70. Bartolucci AA, Tendera M, Howard G: **Meta-analysis of multiple primary prevention trials of cardiovascular events using aspirin**. *Am J Cardiol* 2011, **107**(12):1796-1801.

71. Berger JS, Lala A, Krantz MJ, Baker GS, Hiatt WR: **Aspirin for the prevention of cardiovascular events in patients without clinical cardiovascular disease: a meta-analysis of randomized trials**. *American Heart Journal* 2011, **162**(1):115-124.e112.

72. Butalia S, Leung AA, Ghali WA, Rabi DM: **Aspirin effect on the incidence of major adverse cardiovascular events in patients with diabetes mellitus: a systematic review and meta-analysis**. *Cardiovascular Diabetology* 2011, **10**(1):25.

73. Ebbert J, Montori VM, Erwin PJ, Stead LF: **Interventions for smokeless tobacco use cessation**. In: *Cochrane Database of Systematic Reviews.* edn.: John Wiley & Sons, Ltd; 2011.

74. Ebrahim S, Taylor F, Ward K, Beswick A, Burke M, Davey Smith G: **Multiple risk factor interventions for primary prevention of coronary heart disease**. *Cochrane Database Syst Rev* 2011(1):CD001561.

75. Korczak D, Steinhauser G, Dietl M: **Effectiveness of programmes as part of primary prevention demonstrated on the example of cardiovascular diseases and the metabolic syndrome (Structured abstract)**. In: *Health Technology Assessment Database.* German Agency for Health Technology Assessment at the German Institute for Medical Documentation and Information (DAHTA DIMDI); 2011.

76. Lip GY, Felmeden DC, Dwivedi G: **Antiplatelet agents and anticoagulants for hypertension**. *Cochrane Database of Systematic Reviews* 2011(12):CD003186.

77. Mills EJ, O'Regan C, Eyawo O, Wu P, Mills F, Berwanger O, Briel M: **Intensive statin therapy compared with moderate dosing for prevention of cardiovascular events: a meta-analysis of >40 000 patients**. *European Heart Journal* 2011, **32**(11):1409-1415.

78. Raju N, Sobieraj-Teague M, Hirsh J, O'Donnell M, Eikelboom J: **Effect of aspirin on mortality in the primary prevention of cardiovascular disease**. *Am J Med* 2011, **124**(7):621-629.

79. Stavrakis S, Stoner JA, Azar M, Wayangankar S, Thadani U: **Low-dose aspirin for primary prevention of cardiovascular events in patients with diabetes: a meta-analysis**. *Am J Med Sci* 2011, **341**(1):1-9.

80. Taylor RS, Ashton KE, Moxham T, Hooper L, Ebrahim S: **Reduced dietary salt for the prevention of cardiovascular disease**. *Cochrane Database Syst Rev* 2011(7):Cd009217.

81. Taylor RS, Ashton KE, Moxham T, Hooper L, Ebrahim S: **Reduced dietary salt for the prevention of cardiovascular disease: a meta-analysis of randomized controlled trials (Cochrane review)**. *Am J Hypertens* 2011, **24**(8):843-853.

82. Tonelli M, Lloyd A, Clement F, Conly J, Husereau D, Hemmelgarn B, Klarenbach S, McAlister FA, Wiebe N, Manns B *et al*: **Efficacy of statins for primary prevention in people at low cardiovascular risk: a meta-analysis**. *Cmaj* 2011, **183**(16):E1189-1202.

83. Zhou Y-H, Tang J-Y, Wu M-J, Lu J, Wei X, Qin Y-Y, Wang C, Xu J-F, He J: **Effect of Folic Acid Supplementation on Cardiovascular Outcomes: A Systematic Review and Meta-Analysis**. *PLoS ONE* 2011, **6**(9):e25142.

84. **The effects of lowering LDL cholesterol with statin therapy in people at low risk of vascular disease: meta-analysis of individual data from 27 randomised trials**. *The Lancet* 2012, **380**(9841):581-590.

85. Bjelakovic G, Nikolova D, Gluud Lise L, Simonetti Rosa G, Gluud C: **Antioxidant supplements for prevention of mortality in healthy participants and patients with various diseases**. In: *Cochrane Database of Systematic Reviews.* John Wiley & Sons, Ltd; 2012.

86. Bock C, Diehl K, Schneider S, Diehm C, Litaker D: **Behavioral counseling for cardiovascular disease prevention in primary care settings: a systematic review of practice and associated factors**. *Med Care Res Rev* 2012, **69**(5):495-518.

87. Carey KM, Comee MR, Donovan JL, Kanaan AO: **A polypill for all? Critical review of the polypill literature for primary prevention of cardiovascular disease and stroke**. *Ann Pharmacother* 2012, **46**(5):688-695.

88. Chen YH, Feng B, Chen ZW: **Statins for primary prevention of cardiovascular and cerebrovascular events in diabetic patients without established cardiovascular diseases: a meta-analysis**. *Exp Clin Endocrinol Diabetes* 2012, **120**(2):116-120.

89. de Lorgeril M, Hamazaki T, Kostucki W, Okuyama H, Pavy B, McGill AT, Rabaeus M: **Is the use of cholesterol-lowering drugs for the prevention of cardiovascular complications in type 2 diabetics evidence-based? A systematic review**. *Rev Recent Clin Trials* 2012, **7**(2):150-157.

90. de Vries FM, Denig P, Pouwels KB, Postma MJ, Hak E: **Primary prevention of major cardiovascular and cerebrovascular events with statins in diabetic patients: a meta-analysis**. *Drugs* 2012, **72**(18):2365-2373.

91. Diao D, Wright JM, Cundiff DK, Gueyffier F: **Pharmacotherapy for mild hypertension**. *Cochrane Database of Systematic Reviews* 2012(8):CD006742.

92. Fretheim A, Odgaard-Jensen J, Brors O, Madsen S, Njolstad I, Norheim OF, Svilaas A, Kristiansen IS, Thurmer H, Flottorp S: **Comparative effectiveness of antihypertensive medication for primary prevention of cardiovascular disease: systematic review and multiple treatments meta-analysis**. *BMC Med* 2012, **10**:33.

93. Gutierrez J, Ramirez G, Rundek T, Sacco RL: **Statin therapy in the prevention of recurrent cardiovascular events: a sex-based meta-analysis**. *Archives of Internal Medicine* 2012, **172**(12):909-919.

94. Hooper L, Summerbell CD, Thompson R, Sills D, Roberts FG, Moore HJ, Davey Smith G: **Reduced or modified dietary fat for preventing cardiovascular disease**. *Cochrane Database of Systematic Reviews* 2012(5):CD002137.

95. Minder CM, Blaha MJ, Horne A, Michos ED, Kaul S, Blumenthal RS: **Evidence-based use of statins for primary prevention of cardiovascular disease**. *Am J Med* 2012, **125**(5):440-446.

96. Schnell O, Erbach M, Hummel M: **Primary and secondary prevention of cardiovascular disease in diabetes with aspirin**. *Diab Vasc Dis Res* 2012, **9**(4):245-255.

97. Sepanlou SG, Farzadfar F, Jafari E, Danaei G: **Cardiovascular disease prevention using fixed dose pharmacotherapy in Iran: updated meta-analyses and mortality estimation**. *Arch Iran Med* 2012, **15**(9):531-537.

98. Seshasai SRK: **Effect of Aspirin on Vascular and Nonvascular Outcomes**. *Archives of Internal Medicine* 2012, **172**(3):209.

99. Stabler SN, Tejani AM, Huynh F, Fowkes C: **Garlic for the prevention of cardiovascular morbidity and mortality in hypertensive patients**. *Cochrane Database Syst Rev* 2012, **8**:Cd007653.

100. Tagliabue L, Dipaola F, Perego F, Podda GM, Gruppo di Autoformazione M: **Aspirin for the primary prevention of cardiovascular diseases**. *Intern* 2012, **7**(4):375-379.

101. Valentine N, Van de Laar FA, van Driel ML: **Adenosine-diphosphate (ADP) receptor antagonists for the prevention of cardiovascular disease in type 2 diabetes mellitus**. *Cochrane Database of Systematic Reviews* 2012, **11**:CD005449.

102. Yang HT, Lee M, Hong KS, Ovbiagele B, Saver JL: **Efficacy of folic acid supplementation in cardiovascular disease prevention: an updated meta-analysis of randomized controlled trials**. *Eur* 2012, **23**(8):745-754.

103. Cahill K, Stevens S, Perera R, Lancaster T: **Pharmacological interventions for smoking cessation: an overview and network meta-analysis**. *Cochrane Database of Systematic Reviews* 2013.

104. Chang YH, Hsieh MC, Wang CY, Lin KC, Lee YJ: **Reassessing the benefits of statins in the prevention of cardiovascular disease in diabetic patients--a systematic review and meta-analysis**. *The review of diabetic studies : RDS* 2013, **10**(2-3):157-170.

105. Charan J, Goyal JP, Saxena D: **Effect of Pollypill on cardiovascular parameters: Systematic review and meta-analysis**. *J Cardiovasc Dis Res* 2013, **4**(2):92-97.

106. Fortmann SP, Burda BU, Senger CA, Lin J, Beil T, O'Connor E, Whitlock EP: **Vitamin, mineral, and multivitamin supplements for the primary prevention of cardiovascular disease and cancer (Structured abstract)**. In: *Health Technology Assessment Database.* Agency for Healthcare Research and Quality (AHRQ); 2013.

107. Fortmann SP, Burda BU, Senger CA, Lin JS, Whitlock EP: **Vitamin and mineral supplements in the primary prevention of cardiovascular disease and cancer: An updated systematic evidence review for the U.S. Preventive Services Task Force**. *Annals of internal medicine* 2013, **159**(12):824-834.

108. Hartley L, Flowers N, Holmes J, Clarke A, Stranges S, Hooper L, Rees K: **Green and black tea for the primary prevention of cardiovascular disease**. *Cochrane Database Syst Rev* 2013, **6**:Cd009934.

109. Hartley L, Igbinedion E, Holmes J, Flowers N, Thorogood M, Clarke A, Stranges S, Hooper L, Rees K: **Increased consumption of fruit and vegetables for the primary prevention of cardiovascular diseases**. *Cochrane Database Syst Rev* 2013, **6**:Cd009874.

110. Lavigne PM, Karas RH: **The current state of niacin in cardiovascular disease prevention: a systematic review and meta-regression**. *Journal of the American College of Cardiology* 2013, **61**(4):440-446.

111. Myung SK, Ju W, Cho B, Oh SW, Park SM, Koo BK, Park BJ: **Efficacy of vitamin and antioxidant supplements in prevention of cardiovascular disease: systematic review and meta-analysis of randomised controlled trials**. *Bmj* 2013, **346**:f10.

112. Qin X, Huo Y, Xie D, Hou F, Xu X, Wang X: **Homocysteine-lowering therapy with folic acid is effective in cardiovascular disease prevention in patients with kidney disease: a meta-analysis of randomized controlled trials**. *Clin Nutr* 2013, **32**(5):722-727.

113. Rees K, Dyakova M, Wilson N, Ward K, Thorogood M, Brunner E: **Dietary advice for reducing cardiovascular risk**. *Cochrane Database of Systematic Reviews* 2013(12):CD002128.

114. Rees K, Hartley L, Day C, Flowers N, Clarke A, Stranges S: **Selenium supplementation for the primary prevention of cardiovascular disease**. *Cochrane Database Syst Rev* 2013, **1**:Cd009671.

115. Rees K, Hartley L, Flowers N, Clarke A, Hooper L, Thorogood M, Stranges S: **'Mediterranean' dietary pattern for the primary prevention of cardiovascular disease**. *Cochrane Database Syst Rev* 2013, **8**:Cd009825.

116. Reiner Z: **Statins in the primary prevention of cardiovascular disease**. *Nature reviews Cardiology* 2013, **10**(8):453-464.

117. Sutcliffe P, Connock M, Gurung T, Freeman K, Johnson S, Kandala NB, Grove A, Gurung B, Morrow S, Clarke A: **Aspirin for prophylactic use in the primary prevention of cardiovascular disease and cancer: a systematic review and overview of reviews**. *Health Technol Assess* 2013, **17**(43):1-253.

118. Sutcliffe P, Connock M, Gurung T, Freeman K, Johnson S, Ngianga-Bakwin K, Grove A, Gurung B, Morrow S, Stranges S *et al*: **Aspirin in primary prevention of cardiovascular disease and cancer: a systematic review of the balance of evidence from reviews of randomized trials**. *PLoS One* 2013, **8**(12):e81970.

119. Taylor F, Huffman MD, Macedo AF, Moore THM, Burke M, Davey Smith G, Ward K, Ebrahim S: **Statins for the primary prevention of cardiovascular disease**. *Cochrane Database of Systematic Reviews* 2013.

120. Taylor FC, Huffman M, Ebrahim S: **Statin therapy for primary prevention of cardiovascular disease**. *Jama* 2013, **310**(22):2451-2452.

121. Zhou Y-H, Ye X-F, Yu F-F, Zhang X, Qin Y-Y, Lu J, He J: **Lipid management in the prevention of stroke: a meta-analysis of fibrates for stroke prevention**. *BMC Neurology* 2013, **13**(1).

122. Adler AJ, Taylor F, Martin N, Gottlieb S, Taylor RS, Ebrahim S: **Reduced dietary salt for the prevention of cardiovascular disease**. *Cochrane Database Syst Rev* 2014, **12**:Cd009217.

123. Bjelakovic G, Gluud Lise L, Nikolova D, Whitfield K, Wetterslev J, Simonetti Rosa G, Bjelakovic M, Gluud C: **Vitamin D supplementation for prevention of mortality in adults**. In: *Cochrane Database of Systematic Reviews.* John Wiley & Sons, Ltd; 2014.

124. Brotons C, Benamouzig R, Filipiak KJ, Limmroth V, Borghi C: **A Systematic Review of Aspirin in Primary Prevention: Is It Time for a New Approach?** *American Journal of Cardiovascular Drugs* 2014, **15**(2):113-133.

125. de Cates AN, Farr MR, Wright N, Jarvis MC, Rees K, Ebrahim S, Huffman MD: **Fixed-dose combination therapy for the prevention of cardiovascular disease**. *Cochrane Database Syst Rev* 2014(4):Cd009868.

126. Desai CK, Huang J, Lokhandwala A, Fernandez A, Riaz IB, Alpert JS: **The role of vitamin supplementation in the prevention of cardiovascular disease events**. *Clin Cardiol* 2014, **37**(9):576-581.

127. Flowers N, Hartley L, Todkill D, Stranges S, Rees K: **Co-enzyme Q10 supplementation for the primary prevention of cardiovascular disease**. *Cochrane Database Syst Rev* 2014, **12**:Cd010405.

128. Hartley L, Dyakova M, Holmes J, Clarke A, Lee MS, Ernst E, Rees K: **Yoga for the primary prevention of cardiovascular disease**. *Cochrane Database Syst Rev* 2014, **5**:Cd010072.

129. Hartley L, Flowers N, Lee MS, Ernst E, Rees K: **Tai chi for primary prevention of cardiovascular disease**. *Cochrane Database Syst Rev* 2014, **4**:Cd010366.

130. Kim BH, Cho KI, Jang JS, Park YH, Je HG: **Efficacy and safety of statins for primary prevention of cardiovascular events in women and men: systemic review and up-to-date meta-analysis (Provisional abstract)**. In: *Database of Abstracts of Reviews of Effects.* 2014: 1222-1227.

131. Merriel SW, Andrews V, Salisbury C: **Telehealth interventions for primary prevention of cardiovascular disease: a systematic review and meta-analysis**. *Preventive medicine* 2014, **64**:88-95.

132. Moyer VA: **Vitamin, mineral, and multivitamin supplements for the primary prevention of cardiovascular disease and cancer: U.S. Preventive services Task Force recommendation statement**. *Annals of internal medicine* 2014, **160**(8):558-564.

133. Seron P, Lanas F, Pardo Hernandez H, Bonfill Cosp X: **Exercise for people with high cardiovascular risk**. *Cochrane Database of Systematic Reviews* 2014(8):CD009387.

134. Taylor-Piliae RE: **Tai Ji Quan as an exercise modality to prevent and manage cardiovascular disease: A review**. *Journal of Sport and Health Science* 2014, **3**(1):43-51.

135. Thomopoulos C, Parati G, Zanchetti A: **Effects of blood pressure lowering on outcome incidence in hypertension. 1. Overview, meta-analyses, and meta-regression analyses of randomized trials**. *Journal of Hypertension* 2014, **32**(12):2285-2295.

136. Xie M, Shan Z, Zhang Y, Chen S, Yang W, Bao W, Rong Y, Yu X, Hu FB, Liu L: **Aspirin for primary prevention of cardiovascular events: meta-analysis of randomized controlled trials and subgroup analysis by sex and diabetes status**. *PLoS One* 2014, **9**(10):e90286.

137. **Efficacy and safety of LDL-lowering therapy among men and women: meta-analysis of individual data from 174 000 participants in 27 randomised trials**. *The Lancet* 2015, **385**(9976):1397-1405.

138. Al-Khudairy L, Hartley L, Clar C, Flowers N, Hooper L, Rees K: **Omega 6 fatty acids for the primary prevention of cardiovascular disease**. *Cochrane Database Syst Rev* 2015(11):Cd011094.

139. Boardman HM, Hartley L, Eisinga A, Main C, Roque i Figuls M, Bonfill Cosp X, Gabriel Sanchez R, Knight B: **Hormone therapy for preventing cardiovascular disease in post-menopausal women**. *Cochrane Database of Systematic Reviews* 2015(3):CD002229.

140. Clar C, Oseni Z, Flowers N, Keshtkar-Jahromi M, Rees K: **Influenza vaccines for preventing cardiovascular disease**. *Cochrane Database of Systematic Reviews* 2015(5):CD005050.

141. Desai D, Ahmed HM, Michos ED: **Preventing cardiovascular disease in patients with diabetes: use of aspirin for primary prevention**. *Curr Cardiol Rep* 2015, **17**(3):566.

142. Emdin CA, Rahimi K, Neal B, Callender T, Perkovic V, Patel A: **Blood Pressure Lowering in Type 2 Diabetes**. *Jama* 2015, **313**(6):603.

143. Ettehad D, Emdin CA, Kiran A, Anderson SG, Callender T, Emberson J, Chalmers J, Rodgers A, Rahimi K: **Blood pressure lowering for prevention of cardiovascular disease and death: a systematic review and meta-analysis**. *Lancet (London, England)* 2015.

144. Hartley L, Clar C, Ghannam O, Flowers N, Stranges S, Rees K: **Vitamin K for the primary prevention of cardiovascular disease**. *Cochrane Database Syst Rev* 2015, **9**:Cd011148.

145. Hartley L, Lee MS, Kwong JS, Flowers N, Todkill D, Ernst E, Rees K: **Qigong for the primary prevention of cardiovascular disease**. *Cochrane Database Syst Rev* 2015, **6**:Cd010390.

146. Hooper L, Martin N, Abdelhamid A, Davey Smith G: **Reduction in saturated fat intake for cardiovascular disease**. *Cochrane Database of Systematic Reviews* 2015(6):CD011737.

147. Lipinski MJ, Benedetto U, Escarcega RO, Biondi-Zoccai G, Lhermusier T, Baker NC, Torguson R, Brewer HB, Waksman R: **The impact of proprotein convertase subtilisin-kexin type 9 serine protease inhibitors on lipid levels and outcomes in patients with primary hypercholesterolaemia: a network meta-analysis**. *European Heart Journal* 2015, **37**(6):536-545.

148. Major RW, Cheung CK, Gray LJ, Brunskill NJ: **Statins and Cardiovascular Primary Prevention in CKD: A Meta-Analysis**. *Clin J Am Soc Nephrol* 2015, **10**(5):732-739.

149. Martin N, Germano R, Hartley L, Adler AJ, Rees K: **Nut consumption for the primary prevention of cardiovascular disease**. *Cochrane Database Syst Rev* 2015, **9**:Cd011583.

150. Navarese EP, Kolodziejczak M, Schulze V, Gurbel PA, Tantry U, Lin Y, Brockmeyer M, Kandzari DE, Kubica JM, D'Agostino RB *et al*: **Effects of Proprotein Convertase Subtilisin/Kexin Type 9 Antibodies in Adults With Hypercholesterolemia**. *Annals of internal medicine* 2015, **163**(1):40.

151. Sundström J, Arima H, Jackson R, Turnbull F, Rahimi K, Chalmers J, Woodward M, Neal B: **Effects of Blood Pressure Reduction in Mild Hypertension**. *Annals of internal medicine* 2015, **162**(3):184.

152. Teng M, Lin L, Zhao YJ, Khoo AL, Davis BR, Yong QW, Yeo TC, Lim BP: **Statins for Primary Prevention of Cardiovascular Disease in Elderly Patients: Systematic Review and Meta-Analysis**. *Drugs & aging* 2015, **32**(8):649-661.

153. Uthman OA, Hartley L, Rees K, Taylor F, Ebrahim S, Clarke A: **Multiple risk factor interventions for primary prevention of cardiovascular disease in low- and middle-income countries**. *Cochrane Database Syst Rev* 2015, **8**:Cd011163.

154. Widmer RJ, Collins NM, Collins CS, West CP, Lerman LO, Lerman A: **Digital health interventions for the prevention of cardiovascular disease: a systematic review and meta-analysis**. *Mayo Clinic proceedings* 2015, **90**(4):469-480.

155. Zheng G, Huang M, Liu F, Li S, Tao J, Chen L: **Tai chi chuan for the primary prevention of stroke in middle-aged and elderly adults: a systematic review**. *Evid Based Complement Alternat Med* 2015, **2015**:742152.

156. Cramer H, Langhorst J, Dobos G, Lauche R: **Yoga for metabolic syndrome: A systematic review and meta-analysis**. *European Journal of Preventive Cardiology* 2016, **23**(18):1982-1993.

157. Dyakova M, Shantikumar S, Colquitt JL, Drew CM, Sime M, MacIver J, Wright N, Clarke A, Rees K: **Systematic versus opportunistic risk assessment for the primary prevention of cardiovascular disease**. *Cochrane Database of Systematic Reviews* 2016(1):CD010411.

158. Hartley L, May MD, Loveman E, Colquitt JL, Rees K: **Dietary fibre for the primary prevention of cardiovascular disease**. *Cochrane Database Syst Rev* 2016, **1**:Cd011472.

159. Hemkens LG, Ewald H, Gloy VL, Arpagaus A, Olu KK, Nidorf M, Glinz D, Nordmann AJ, Briel M: **Colchicine for prevention of cardiovascular events**. *Cochrane Database Syst Rev* 2016(1):Cd011047.

160. Jakob T, Nordmann AJ, Schandelmaier S, Ferreira-Gonzalez I, Briel M: **Fibrates for primary prevention of cardiovascular disease events**. *Cochrane Database of Systematic Reviews* 2016, **11**:CD009753.

161. Kelly J, Khalesi S, Dickinson K, Hines S, Coombes JS, Todd AS: **The effect of dietary sodium modification on blood pressure in adults with systolic blood pressure less than 140 mmHg: a systematic review**. *JBI Database System Rev Implement Rep* 2016, **14**(6):196-237.

162. Lei H, Gao Q, Liu SR, Xu J: **The Benefit and Safety of Aspirin for Primary Prevention of Ischemic Stroke: A Meta-Analysis of Randomized Trials**. *Front Pharmacol* 2016, **7**:440.

163. Li Y, Huang T, Zheng Y, Muka T, Troup J, Hu FB: **Folic Acid Supplementation and the Risk of Cardiovascular Diseases: A Meta-Analysis of Randomized Controlled Trials**. *J Am Heart Assoc* 2016, **5**(8):15.

164. Padwal R, Hackam D, Khan N, Tobe S: **Primary prevention of CVD: modification of diet in people with hypertension**. *Clin Evid (Online)* 2016, **05**:05.

165. Walz CP, Barry AR, Koshman SL: **Omega-3 polyunsaturated fatty acid supplementation in the prevention of cardiovascular disease**. *Can Pharm J (Ott)* 2016, **149**(3):166-173.

166. Wu JH, Foote C, Blomster J, Toyama T, Perkovic V, Sundstrom J, Neal B: **Effects of sodium-glucose cotransporter-2 inhibitors on cardiovascular events, death, and major safety outcomes in adults with type 2 diabetes: a systematic review and meta-analysis**. *Lancet Diabetes Endocrinol* 2016, **4**(5):411-419.

167. Xie X, Atkins E, Lv J, Bennett A, Neal B, Ninomiya T, Woodward M, MacMahon S, Turnbull F, Hillis GS *et al*: **Effects of intensive blood pressure lowering on cardiovascular and renal outcomes: updated systematic review and meta-analysis**. *The Lancet* 2016, **387**(10017):435-443.

168. Al-Khudairy L, Flowers N, Wheelhouse R, Ghannam O, Hartley L, Stranges S, Rees K: **Vitamin C supplementation for the primary prevention of cardiovascular disease**. In: *Cochrane Database of Systematic Reviews.* John Wiley & Sons, Ltd; 2017.

169. Alageel S, Gulliford MC, McDermott L, Wright AJ: **Multiple health behaviour change interventions for primary prevention of cardiovascular disease in primary care: systematic review and meta-analysis**. *BMJ Open* 2017, **7**(6):e015375.

170. Alhassan A, Young J, Lean MEJ, Lara J: **Consumption of fish and vascular risk factors: A systematic review and meta-analysis of intervention studies**. *Atherosclerosis* 2017, **266**:87-94.

171. Bahiru E, de Cates AN, Farr MR, Jarvis MC, Palla M, Rees K, Ebrahim S, Huffman MD: **Fixed-dose combination therapy for the prevention of atherosclerotic cardiovascular diseases**. *Cochrane Database of Systematic Reviews* 2017, **3**:CD009868.

172. Cheng HM, Koutsidis G, Lodge JK, Ashor A, Siervo M, Lara J: **Tomato and lycopene supplementation and cardiovascular risk factors: A systematic review and meta-analysis**. *Atherosclerosis* 2017, **257**:100-108.

173. Cheong AT, Liew SM, Khoo EM, Mohd Zaidi NF, Chinna K: **Are interventions to increase the uptake of screening for cardiovascular disease risk factors effective? A systematic review and meta-analysis**. *BMC Fam Pract* 2017, **18**(1):4.

174. Clar C, Al-Khudairy L, Loveman E, Kelly SA, Hartley L, Flowers N, Germano R, Frost G, Rees K: **Low glycaemic index diets for the prevention of cardiovascular disease**. *Cochrane Database of Systematic Reviews* 2017, **7**:CD004467.

175. Hartley L, Mavrodaris A, Flowers N, Ernst E, Rees K: **Transcendental meditation for the primary prevention of cardiovascular disease**. In: *Cochrane Database of Systematic Reviews.* John Wiley & Sons, Ltd; 2017.

176. Jeet G, Thakur JS, Prinja S, Singh M: **Community health workers for non-communicable diseases prevention and control in developing countries: Evidence and implications**. *PLoS ONE* 2017, **12**(7):e0180640.

177. Karmali KN, Persell SD, Perel P, Lloyd-Jones DM, Berendsen MA, Huffman MD: **Risk scoring for the primary prevention of cardiovascular disease**. *Cochrane Database of Systematic Reviews* 2017, **3**:CD006887.

178. Kelly SA, Hartley L, Loveman E, Colquitt JL, Jones HM, Al-Khudairy L, Clar C, Germano R, Lunn HR, Frost G *et al*: **Whole grain cereals for the primary or secondary prevention of cardiovascular disease**. *Cochrane Database of Systematic Reviews* 2017, **8**:CD005051.

179. Kunutsor SK, Seidu S, Khunti K: **Aspirin for primary prevention of cardiovascular and all-cause mortality events in diabetes: updated meta-analysis of randomized controlled trials**. *Diabet Med* 2017, **34**(3):316-327.

180. Lauche R, Peng W, Ferguson C, Cramer H, Frawley J, Adams J, Sibbritt D: **Efficacy of Tai Chi and qigong for the prevention of stroke and stroke risk factors: A systematic review with meta-analysis**. *Medicine (Baltimore)* 2017, **96**(45):e8517.

181. Marti-Carvajal AJ, Sola I, Lathyris D, Dayer M: **Homocysteine-lowering interventions for preventing cardiovascular events**. *Cochrane Database of Systematic Reviews* 2017, **8**:CD006612.

182. Mosdøl A, Lidal Ingeborg B, Straumann Gyri H, Vist Gunn E: **Targeted mass media interventions promoting healthy behaviours to reduce risk of non-communicable diseases in adult, ethnic minorities**. In: *Cochrane Database of Systematic Reviews.* John Wiley & Sons, Ltd; 2017.

183. Nunes JP: **Statins in primary prevention: impact on mortality. A meta-analysis study**. *Minerva Cardioangiol* 2017, **65**(5):531-538.

184. Patnode CD, Evans CV, Senger CA, Redmond N, Lin JS: *Agency for Healthcare Research and Quality (US)* 2017:07.

185. Ramoa Castro A, Oliveira NL, Ribeiro F, Oliveira J: **Impact of educational interventions on primary prevention of cardiovascular disease: A systematic review with a focus on physical activity**. *Eur J Gen Pract* 2017, **23**(1):59-68.

186. Reed JL, Prince SA, Elliott CG, Mullen KA, Tulloch HE, Hiremath S, Cotie LM, Pipe AL, Reid RD: **Impact of Workplace Physical Activity Interventions on Physical Activity and Cardiometabolic Health Among Working-Age Women: A Systematic Review and Meta-Analysis**. *Circ Cardiovasc Qual Outcomes* 2017, **10**(2).

187. Rizos EC, Elisaf MS: **Does Supplementation with Omega-3 PUFAs Add to the Prevention of Cardiovascular Disease?** *Curr Cardiol Rep* 2017, **19**(6):47.

188. Schandelmaier S, Briel M, Saccilotto R, Olu KK, Arpagaus A, Hemkens LG, Nordmann AJ: **Niacin for primary and secondary prevention of cardiovascular events**. *Cochrane Database of Systematic Reviews* 2017, **6**:CD009744.

189. Schmidt AF, Pearce LS, Wilkins JT, Overington JP, Hingorani AD, Casas JP: **PCSK9 monoclonal antibodies for the primary and secondary prevention of cardiovascular disease**. *Cochrane Database of Systematic Reviews* 2017, **4**:CD011748.

190. Schwingshackl L, Boeing H, Stelmach-Mardas M, Gottschald M, Dietrich S, Hoffmann G, Chaimani A: **Dietary Supplements and Risk of Cause-Specific Death, Cardiovascular Disease, and Cancer: A Systematic Review and Meta-Analysis of Primary Prevention Trials**. *Adv Nutr (Bethesda)* 2017, **8**(1):27-39.

191. Squizzato A, Bellesini M, Takeda A, Middeldorp S, Donadini MP: **Clopidogrel plus aspirin versus aspirin alone for preventing cardiovascular events**. *Cochrane Database of Systematic Reviews* 2017, **12**:CD005158.

192. Tomasik T, Krzyszton J, Dubas-Jakobczyk K, Kijowska V, Windak A: **The systematic coronary risk evaluation (SCORE) for the prevention of cardiovascular diseases. Does evidence exist for its effectiveness? A systematic review**. *Acta Cardiologica* 2017, **72**(4):370-379.

193. Wang WW, Wang XS, Zhang ZR, He JC, Xie CL: **A Meta-Analysis of Folic Acid in Combination with Anti-Hypertension Drugs in Patients with Hypertension and Hyperhomocysteinemia**. *Front Pharmacol* 2017, **8**:585.

194. Watanabe Y, Tatsuno I: **Omega-3 polyunsaturated fatty acids for cardiovascular diseases: present, past and future**. *Expert Rev Clin Pharmacol* 2017, **10**(8):865-873.

195. Wei CY, Quek RG, Villa G, Gandra SR, Forbes CA, Ryder S, Armstrong N, Deshpande S, Duffy S, Kleijnen J *et al*: **A Systematic Review of Cardiovascular Outcomes-Based Cost-Effectiveness Analyses of Lipid-Lowering Therapies**. *Pharmacoeconomics* 2017, **35**(3):297-318.

196. Yang L, Ling W, Du Z, Chen Y, Li D, Deng S, Liu Z, Yang L: **Effects of Anthocyanins on Cardiometabolic Health: A Systematic Review and Meta-Analysis of Randomized Controlled Trials**. *Adv Nutr (Bethesda)* 2017, **8**(5):684-693.

197. Zhang Y, Sun N, Jiang X, Xi Y: **Comparative efficacy of beta-blockers on mortality and cardiovascular outcomes in patients with hypertension: a systematic review and network meta-analysis**. *J Am Soc Hypertens* 2017, **11**(7):394-401.

198. Abdelhamid AS, Brown TJ, Brainard JS, Biswas P, Thorpe GC, Moore HJ, Deane KH, AlAbdulghafoor FK, Summerbell CD, Worthington HV *et al*: **Omega-3 fatty acids for the primary and secondary prevention of cardiovascular disease**. *Cochrane Database of Systematic Reviews* 2018, **7**:CD003177.

199. Abdelhamid AS, Martin N, Bridges C, Brainard JS, Wang X, Brown TJ, Hanson S, Jimoh OF, Ajabnoor SM, Deane KH *et al*: **Polyunsaturated fatty acids for the primary and secondary prevention of cardiovascular disease**. *Cochrane Database of Systematic Reviews* 2018, **7**:CD012345.

200. Al-Ghamdi S: **The association between olive oil consumption and primary prevention of cardiovascular diseases**. *J* 2018, **7**(5):859-864.

201. Brouwer TF, Vehmeijer JT, Kalkman DN, Berger WR, van den Born BH, Peters RJ, Knops RE: **Intensive Blood Pressure Lowering in Patients With and Patients Without Type 2 Diabetes: A Pooled Analysis From Two Randomized Trials**. *Diabetes Care* 2018, **41**(6):1142-1148.

202. Brunstrom M, Carlberg B: **Association of Blood Pressure Lowering With Mortality and Cardiovascular Disease Across Blood Pressure Levels: A Systematic Review and Meta-analysis**. *JAMA Intern Med* 2018, **178**(1):28-36.

203. Chiavaroli L, Nishi SK, Khan TA, Braunstein CR, Glenn AJ, Mejia SB, Rahelic D, Kahleova H, Salas-Salvado J, Jenkins DJA *et al*: **Portfolio Dietary Pattern and Cardiovascular Disease: A Systematic Review and Meta-analysis of Controlled Trials**. *Prog Cardiovasc Dis* 2018, **61**(1):43-53.

204. Cui JY, Zhou RR, Han S, Wang TS, Wang LQ, Xie XH: **Statin therapy on glycemic control in type 2 diabetic patients: A network meta-analysis**. *J Clin Pharm Ther* 2018, **43**(4):556-570.

205. Dinu M, Pagliai G, Casini A, Sofi F: **Mediterranean diet and multiple health outcomes: an umbrella review of meta-analyses of observational studies and randomised trials**. *European Journal of Clinical Nutrition* 2018, **72**(1):30-43.

206. Fei Y, Guyatt GH, Alexander PE, El Dib R, Siemieniuk RAC, Vandvik PO, Nunnally ME, Gomaa H, Morgan RL, Agarwal A *et al*: **Addition of Ezetimibe to statins for patients at high cardiovascular risk: Systematic review of patient-important outcomes**. *J Eval Clin Pract* 2018, **24**(1):222-231.

207. Guasch-Ferre M, Li J, Hu FB, Salas-Salvado J, Tobias DK: **Effects of walnut consumption on blood lipids and other cardiovascular risk factors: an updated meta-analysis and systematic review of controlled trials**. *Am J Clin Nutr* 2018, **108**(1):174-187.

208. Hong Z, Wu T, Zhou S, Huang B, Wang J, Jin D, Geng D: **Effects of anti-hypertensive treatment on major cardiovascular events in populations within prehypertensive levels: a systematic review and meta-analysis**. *J Hum Hypertens* 2018, **32**(2):94-104.

209. Hooper L, Al‐Khudairy L, Abdelhamid AS, Rees K, Brainard JS, Brown TJ, Ajabnoor SM, O'Brien AT, Winstanley LE, Donaldson DH *et al*: **Omega‐6 fats for the primary and secondary prevention of cardiovascular disease**. *Cochrane Database of Systematic Reviews* 2018(11).

210. Karlson BW, Nicholls SJ, Lundman P, Barter PJ, Palmer MK: **Modeling Statin-Induced Reductions of Cardiovascular Events in Primary Prevention: A VOYAGER Meta-Analysis**. *Cardiology* 2018, **140**(1):30-34.

211. Karmali KN, Lloyd-Jones DM, van der Leeuw J, Goff DC, Jr., Yusuf S, Zanchetti A, Glasziou P, Jackson R, Woodward M, Rodgers A *et al*: **Blood pressure-lowering treatment strategies based on cardiovascular risk versus blood pressure: A meta-analysis of individual participant data**. *PLoS Med* 2018, **15**(3):e1002538.

212. Kim J, Choi J, Kwon SY, McEvoy JW, Blaha MJ, Blumenthal RS, Guallar E, Zhao D, Michos ED: **Association of Multivitamin and Mineral Supplementation and Risk of Cardiovascular Disease: A Systematic Review and Meta-Analysis**. *Circ Cardiovasc Qual Outcomes* 2018, **11**(7):e004224.

213. Leshno M, Goldbourt U, Pinchuk I, Lichtenberg D: **The cardiovascular benefits of indiscriminate supplementation of omega-3 fatty acids; meta-analysis and decision-making approach**. *Int J Food Sci Nutr* 2018, **69**(5):549-556.

214. Nayak A, Hayen A, Zhu L, McGeechan K, Glasziou P, Irwig L, Doust J, Gregory G, Bell K: **Legacy effects of statins on cardiovascular and all-cause mortality: a meta-analysis**. *BMJ Open* 2018, **8**(9):e020584.

215. Oja P, Kelly P, Murtagh EM, Murphy MH, Foster C, Titze S: **Effects of frequency, intensity, duration and volume of walking interventions on CVD risk factors: a systematic review and meta-regression analysis of randomised controlled trials among inactive healthy adults**. *BJSM online* 2018, **52**(12):769-775.

216. Palmer MJ, Barnard S, Perel P, Free C: **Mobile phone-based interventions for improving adherence to medication prescribed for the primary prevention of cardiovascular disease in adults**. *Cochrane Database of Systematic Reviews* 2018, **6**:CD012675.

217. Patil SJ, Ruppar T, Koopman RJ, Lindbloom EJ, Elliott SG, Mehr DR, Conn VS: **Effect of peer support interventions on cardiovascular disease risk factors in adults with diabetes: a systematic review and meta-analysis**. *BMC Public Health* 2018, **18**(1):398.

218. Peterson SC, Barry AR: **Effect of Glucagon-like Peptide-1 Receptor Agonists on All-cause Mortality and Cardiovascular Outcomes: A Meta-analysis**. *Curr Diabetes Rev* 2018, **14**(3):273-279.

219. Sisti LG, Dajko M, Campanella P, Shkurti E, Ricciardi W, de Waure C: **The effect of multifactorial lifestyle interventions on cardiovascular risk factors: a systematic review and meta-analysis of trials conducted in the general population and high risk groups**. *Prev Med* 2018, **109**:82-97.

220. Suh DC, Griggs SK, Henderson ER, Lee SM, Park T: **Comparative effectiveness of lipid-lowering treatments to reduce cardiovascular disease**. *Expert rev* 2018, **18**(1):51-69.

221. Wilson PWF, Polonsky TS, Miedema MD, Khera A, Kosinski AS, Kuvin JT: **Systematic Review for the 2018 AHA/ACC/AACVPR/AAPA/ABC/ACPM/ADA/AGS/APhA/ASPC/NLA/PCNA Guideline on the Management of Blood Cholesterol**. *Circulation* 2018:CIR0000000000000626.

222. Zhan S, Tang M, Liu F, Xia P, Shu M, Wu X: **Ezetimibe for the prevention of cardiovascular disease and all-cause mortality events**. *Cochrane Database of Systematic Reviews* 2018, **11**:CD012502.

223. Zhang B, Zhao Q, Guo W, Bao W, Wang X: **Association of whole grain intake with all-cause, cardiovascular, and cancer mortality: a systematic review and dose-response meta-analysis from prospective cohort studies**. *European Journal of Clinical Nutrition* 2018, **72**(1):57-65.

224. Zhang X, Liu Y, Zhang F, Li J, Tong N: **Legacy Effect of Intensive Blood Glucose Control on Cardiovascular Outcomes in Patients With Type 2 Diabetes and Very High Risk or Secondary Prevention of Cardiovascular Disease: A Meta-analysis of Randomized Controlled Trials**. *Clin Ther* 2018, **40**(5):776-788.e773.

225. Al-Sofiani ME, Derenbecker R, Quartuccio M, Kalyani RR: **Aspirin for Primary Prevention of Cardiovascular Disease in Diabetes: a Review of the Evidence**. *Current diabetes reports* 2019, **19**(10):107.

226. Barbarawi M, Kheiri B, Zayed Y, Gakhal I, Al-Abdouh A, Barbarawi O, Rashdan L, Rizk F, Bachuwa G, Alkotob ML: **Aspirin Efficacy in Primary Prevention: A Meta-analysis of Randomized Controlled Trials**. *High blood pressure & cardiovascular prevention : the official journal of the Italian Society of Hypertension* 2019, **26**(4):283-291.

227. Chiavaroli L, Viguiliouk E, Nishi SK, Blanco Mejia S, Rahelic D, Kahleova H, Salas-Salvado J, Kendall CW, Sievenpiper JL: **DASH Dietary Pattern and Cardiometabolic Outcomes: An Umbrella Review of Systematic Reviews and Meta-Analyses**. *Nutrients* 2019, **11**(2).

228. Christiansen M, Grove EL, Hvas AM: **Primary Prevention of Cardiovascular Events with Aspirin: Toward More Harm than Benefit-A Systematic Review and Meta-Analysis**. *Semin Thromb Hemost* 2019, **45**(5):478-489.

229. Dykun I, Mincu R, Hendricks S, Balcer B, Totzeck M, Rassaf T, Mahabadi AA: **Efficacy of lipid-lowering therapy beyond statins to prevent cardiovascular events: a meta-analysis**. *European journal of preventive cardiology* 2019:2047487319866992.

230. Judge C, Ruttledge S, Murphy R, Loughlin E, Gorey S, Costello M, Nolan A, Ferguson J, Halloran MO, O'Canavan M *et al*: **Aspirin for primary prevention of stroke in individuals without cardiovascular disease-A meta-analysis**. *International journal of stroke : official journal of the International Stroke Society* 2019:1747493019858780.

231. Khan SU, Ul Abideen Asad Z, Khan MU, Talluri S, Ali F, Shahzeb Khan M, Lone AN, Mookadam F, Krasuski RA, Kaluski E: **Aspirin for primary prevention of cardiovascular outcomes in diabetes mellitus: An updated systematic review and meta-analysis**. *European journal of preventive cardiology* 2019:2047487319825510.

232. Krogsboll LT, Jorgensen KJ, Gotzsche PC: **General health checks in adults for reducing morbidity and mortality from disease**. *The Cochrane database of systematic reviews* 2019, **1**:CD009009.

233. Li M, Wang X, Li X, Chen H, Hu Y, Zhang X, Tang X, Miao Y, Tian G, Shang H: **Statins for the Primary Prevention of Coronary Heart Disease**. *BioMed Research International* 2019, **2019**:4870350.

234. Lin M-H, Lee C-H, Lin C, Zou Y-F, Lu C-H, Hsieh C-H, Lee C-H: **Low-Dose Aspirin for the Primary Prevention of Cardiovascular Disease in Diabetic Individuals: A Meta-Analysis of Randomized Control Trials and Trial Sequential Analysis**. *Journal of clinical medicine* 2019, **8**(5).

235. Lu Z, Liu C, Pang X, Dong S: **An update meta-analysis of antiplatelet therapy in high risk patients for primary prevention**. *Acta Cardiologica* 2019, **74**(Supplement 1):62.

236. Mahmoud AN, Gad MM, Elgendy AY, Elgendy IY, Bavry AA: **Efficacy and safety of aspirin for primary prevention of cardiovascular events: a meta-analysis and trial sequential analysis of randomized controlled trials**. *European heart journal* 2019, **40**(7):607-617.

237. Miao XY, Liu HZ, Jin MM, Sun BR, Tian H, Li J, Li N, Yan ST: **A comparative meta-analysis of the efficacy of statin-ezetimibe co-therapy versus statin monotherapy in reducing cardiovascular and cerebrovascular adverse events in patients with type 2 diabetes mellitus**. *European Review for Medical and Pharmacological Sciences* 2019, **23**(5):2302-2310.

238. Milionis H, Ntaios G, Korompoki E, Vemmos K, Michel P: **Statin-based therapy for primary and secondary prevention of ischemic stroke: A meta-analysis and critical overview**. *International journal of stroke : official journal of the International Stroke Society* 2019:1747493019873594.

239. Musini VM, Tejani AM, Bassett K, Puil L, Wright JM: **Pharmacotherapy for hypertension in adults 60 years or older**. *The Cochrane database of systematic reviews* 2019, **6**:CD000028.

240. Rees K, Takeda A, Martin N, Ellis L, Wijesekara D, Vepa A, Das A, Hartley L, Stranges S: **Mediterranean-style diet for the primary and secondary prevention of cardiovascular disease**. *The Cochrane database of systematic reviews* 2019, **3**:CD009825.

241. Seidu S, Kunutsor SK, Sesso HD, Gaziano JM, Buring JE, Roncaglioni MC, Khunti K: **Aspirin has potential benefits for primary prevention of cardiovascular outcomes in diabetes: updated literature-based and individual participant data meta-analyses of randomized controlled trials**. *Cardiovascular diabetology* 2019, **18**(1):70.

242. Shah R, Khan B, Latham SB, Khan SA, Rao SV: **A Meta-Analysis of Aspirin for the Primary Prevention of Cardiovascular Diseases in the Context of Contemporary Preventive Strategies**. *The American journal of medicine* 2019.

243. Shah R, Rawal A, Najib K, Khan B, Hesterberg K, Rashid A, Latham SB: **ASPIRIN FOR THE PRIMARY PREVENTION OF CARDIOVASCULAR EVENTS: AN UPDATED META-ANALYSIS OF RANDOMIZED CLINICAL TRIALS**. *Journal of the American College of Cardiology* 2019, **73**(9 Supplement 1):1782.

244. Studzinski K, Tomasik T, Krzyszton J, Jozwiak J, Windak A: **Effect of using cardiovascular risk scoring in routine risk assessment in primary prevention of cardiovascular disease: an overview of systematic reviews**. *BMC Cardiovasc Disord* 2019, **19**(1):11.

245. Viguiliouk E, Kendall CW, Kahleova H, Rahelic D, Salas-Salvado J, Choo VL, Mejia SB, Stewart SE, Leiter LA, Jenkins DJ *et al*: **Effect of vegetarian dietary patterns on cardiometabolic risk factors in diabetes: A systematic review and meta-analysis of randomized controlled trials**. *Clin Nutr* 2019, **38**(3):1133-1145.

246. Xie W, Luo Y, Liang X, Lin Z, Wang Z, Liu M: **The Efficacy And Safety Of Aspirin As The Primary Prevention Of Cardiovascular Disease: An Updated Meta-Analysis**. *Therapeutics and clinical risk management* 2019, **15**:1129-1140.

247. Yebyo HG, Aschmann HE, Kaufmann M, Puhan MA: **Comparative effectiveness and safety of statins as a class and of specific statins for primary prevention of cardiovascular disease: A systematic review, meta-analysis, and network meta-analysis of randomized trials with 94,283 participants**. *American heart journal* 2019, **210**:18-28.

248. Zelniker TA, Wiviott SD, Raz I, Im K, Goodrich EL, Furtado RHM, Bonaca MP, Mosenzon O, Kato ET, Cahn A *et al*: **Comparison of the Effects of Glucagon-Like Peptide Receptor Agonists and Sodium-Glucose Cotransporter 2 Inhibitors for Prevention of Major Adverse Cardiovascular and Renal Outcomes in Type 2 Diabetes Mellitus**. *Circulation* 2019, **139**(17):2022-2031.

249. Zheng SL, Roddick AJ: **Association of Aspirin Use for Primary Prevention With Cardiovascular Events and Bleeding Events: A Systematic Review and Meta-analysis**. *Jama* 2019, **321**(3):277-287.

**Additional File 1:**
