# Supplementary material for: Determining optimal strategies for primary prevention of cardiovascular disease: systematic review, cost-effectiveness review and network meta-analysis protocol
Source: Syst Rev. 2020 May 7;9:105. doi: 10.1186/s13643-020-01366-x (PMC7204030; doi:10.1186/s13643-020-01366-x)
Supplement: Supplementary file 3 — Additional file 3. Cochrane CENTRAL search strategy [file 13643_2020_1366_MOESM3_ESM.docx]

**CENTRAL (Wiley)**

ID Search Hits

#1 MeSH descriptor: [Primary Prevention] explode all trees

#2 "primary prevention":ti,ab,kw

#3 #1 or #2

#4 MeSH descriptor: [Cardiovascular Diseases] explode all trees

#5 MeSH descriptor: [Stroke] explode all trees

#6 (CVD or cardiovascular* or coronary* or heart* or myocardial* or cardiac* or stroke* or cerebrovascular or atherosclerosis or arteriosclerosis or vascular or hypertension or "blood pressure" or cholesterol or lipid*):ti,ab,kw

#7 #4 or #5 or #6

#8 #3 and #7

#9 [mh ^"cardiovascular diseases"/PC]

#10 [mh "coronary disease"/PC]

#11 [mh "myocardial ischemia"/PC]

#12 [mh "heart failure"/PC]

#13 [mh "heart arrest"/PC]

#14 [mh "stroke"/PC]

#15 [mh "carotid stenosis"/PC]

#16 [mh "arteriosclerosis"/PC]

#17 #9 or #10 or #11 or #12 or #13 or #14 or #15 or #16

#18 ((prevent* or reduc* or lower* or decreas* or change* or effect or effects or progression or level* or incidence) near/10 (CVD or cardiovascular* or coronary* or heart* or myocardial or cardiac* or stroke* or cerebrovascular or atherosclerosis or arteriosclerosis or vascular or hypertension or "blood pressure" or cholesterol or lipid*)):ti in Trials
